# Association Between Reporting Antimicrobial Use and *Clostridioides difficile* Standardized Infection Ratios in South Carolina Hospitals

**DOI:** 10.3390/pharmacy13020033

**Published:** 2025-02-22

**Authors:** Maya Abo-Hamzy, Kayla Antosz, Sarah E. Battle, Pamela Bailey, Hana R. Winders, P. Brandon Bookstaver, Majdi N. Al-Hasan

**Affiliations:** 1 Medical University of South Carolina, Charleston, SC 29425, USA; 2 Department of Clinical Pharmacy and Outcomes Sciences, University of South Carolina College of Pharmacy, Columbia, SC 29208, USA; kantosz@mailbox.sc.edu (K.A.); bookstaver@cop.sc.edu (P.B.B.); 3 Department of Internal Medicine, Prisma Health Midlands, Columbia, SC 29203, USA; sarah.cain@prismahealth.org (S.E.B.); pamela.bailey@uscmed.sc.edu (P.B.); 4 Department of Medicine, University of South Carolina School of Medicine, Columbia, SC 29209, USA; 5 Department of Pharmacy, Prisma Health Midlands, Columbia, SC 29203, USA; hana.winders@prismahealth.org

**Keywords:** antimicrobial stewardship, antibiotics, metrics, *C. difficile*, hospital-acquired infections, days of therapy

## Abstract

The Centers for Disease Control and Prevention have been encouraging hospitals in the United States to report antimicrobial use (AU) to the National Healthcare Safety Network (NHSN). This retrospective cohort study examines the association between reporting AU and the *Clostridioides difficile* infection (CDI) standardized infection ratio (SIR) in South Carolina hospitals. Student’s *t*-test was used to examine the mean difference in the change in CDI SIRs from 2017 to 2021 between hospitals reporting AU for ≥3 years and those reporting AU for <3 years during the study period. Among 65 hospitals in South Carolina, 43 reported AU for <3 years, and 22 reported AU for ≥3 years. There was significantly greater decline in the CDI SIR from 2017 to 2021 in hospitals reporting AU for ≥3 years compared to those reporting AU for <3 years (mean difference of the change in the CDI SIR −0.33 [95% CI −0.57, −0.06]; *p* = 0.016). The results of a steeper decline in the CDI SIR in hospitals consistently reporting AU during the majority of the study period compared to other hospitals encourages hospitals to report AU to the NHSN and promotes antimicrobial stewardship efforts at the state and national level.

## 1. Introduction

*Clostridioides difficile* infection (CDI) is one of the most common hospital-acquired infections, with the risk increasing eight- to ten-fold in association with antimicrobial use [1]. Antimicrobial stewardship programs optimize the prescribing of antimicrobial agents, which includes reduction of excessive use. Antimicrobial use (AU), frequently measured in days of therapy, is considered the most direct antimicrobial stewardship metric in hospitals [2]. Interpretation of AU data, however, can be limited by facility-level variability in hospital characteristics and the complexity of patient populations. The Centers for Disease Control and Prevention’s (CDC) National Healthcare Safety Network (NHSN) standardized AU across hospitals in the United States by providing a standardized antimicrobial administration ratio (SAAR) that allows for interfacility comparisons [3]. Since 2016, the Antimicrobial Stewardship Collaborative of South Carolina (ASC-SC) has led statewide efforts encouraging hospitals to participate in AU reporting. However, the potential benefits of reporting AU to individual hospitals remain unclear. This retrospective, multicenter cohort study examined the impact of AU reporting to the NHSN on hospital-onset CDI standardized infection ratios (SIRs).

## 2. Materials and Methods

The ASC-SC was established in 2016 as a collaboration among public health, academic medical centers, and acute care hospitals in South Carolina [4]. The main goals of ASC-SC were to advance antimicrobial stewardship practice and education, improve the appropriateness and reporting of AU in acute care and ambulatory settings, and extend expertise and resources to rural hospitals and skilled nursing facilities in the state. During the study period, ASC-SC held annual statewide meetings, annual regional meetings focusing on the four geographical areas in the state between 2017 and 2019, and bi-weekly virtual meetings with hospitals and ambulatory partners starting in 2020. The purpose of these various meetings was to exchange best antimicrobial stewardship practices, provide antimicrobial stewardship education, support the implementation of antimicrobial stewardship’s core elements, and encourage and facilitate reporting of AU by acute care hospitals to the NHSN. In addition, ASC-SC started sending out quarterly comparative SAAR analysis reports to hospitals reporting AU in 2019, providing statewide comparisons of SAARs and site-specific feedback [5].

This study utilized AU records for hospitals reporting to the NHSN through the South Carolina Department of Public Health (previously the Department of Health and Environmental Control) and publicly reported CDI SIRs from 2017 to 2021 in all hospitals in South Carolina [6]. Hospitals were categorized into those reporting AU during the majority of the 5-year study period (≥3 years) and those reporting AU for <3 years, including those that did not report AU to the NHSN. All reporting hospitals reported AU in all SAAR categories, as defined by the NHSN (overall SAAR, broad-spectrum antibacterial agents for hospital-onset and community-acquired infections, narrow-spectrum beta-lactam agents, antibacterial agents predominantly used for resistant Gram-positive infections, etc.) [7]. The study outcome was a change in the CDI SIR from 2017 to 2021 in each hospital. A matched pairs mean difference was calculated for hospitals that had a publicly reported CDI SIR in both 2017 and 2021. Student’s *t*-test was used to examine the difference in the change in CDI SIRs from 2017 to 2021 between hospitals reporting AU for ≥3 years and those reporting AU for <3 years. Mean differences with 95% confidence intervals (CIs) were reported to define the change in the CDI SIR between 2017 and 2021. Statistical significance was defined as a *p*-value < 0.05. JMP Pro version 17 (Carey, NC, USA) was used for statistical analysis.

## 3. Results

**Figure 1:**
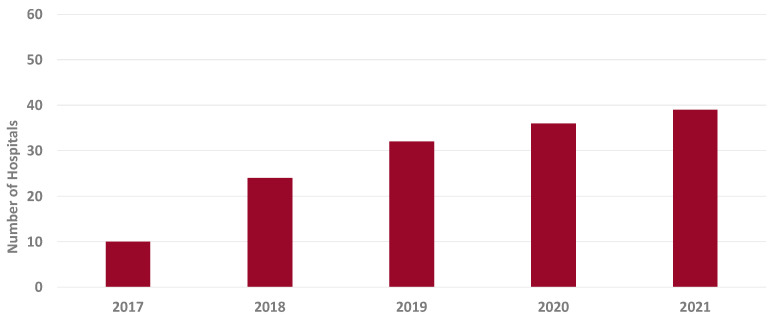
Number of hospitals in South Carolina reporting antimicrobial use to the National Healthcare Safety Network between 2017 and 2021.

Seventy-four hospitals in South Carolina had a publicly reported CDI SIR in any of the 5 years of the study. The number of hospitals reporting AU to the NHSN increased from 10 to 39 between 2017 and 2021 (Figure 1). To describe the level of stewardship for hospitals in South Carolina during the study period, 87% of hospitals in South Carolina implemented all seven of the CDC’s core elements in 2017, and 99% of hospitals implemented all elements in 2021 [8].

**Figure 2:**
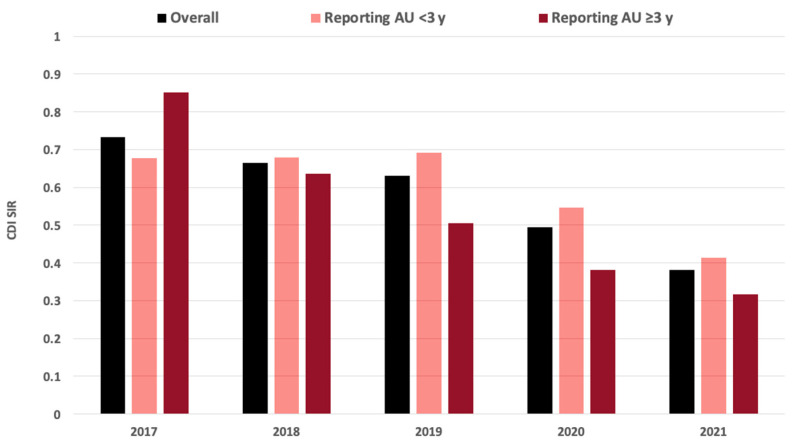
*Clostridioides difficile* infection standardized infection ratios (CDI SIRs) in South Carolina hospitals in 2017–2021 by antimicrobial use (AU) reporting.

Overall, the CDI SIR declined from 0.73 to 0.38 between 2017 and 2021 in the 74 hospitals in South Carolina (Figure 2). Of those, 65 hospitals had publicly reported CDI SIR in both 2017 and 2021. Among these 65 hospitals, the CDI SIR declined from 0.76 in 2017 to 0.41 in 2021 (mean difference −0.34 [95% CI −0.47, −0.21]; *p* < 0.001).

Fifty hospitals reported AU to the NHSN for <3 years during the study period. Forty-three of those hospitals had CDI SIR data available for both 2017 and 2021. The CDI SIR declined from 0.69 to 0.46 between 2017 and 2021 (mean difference −0.23 [95% CI −0.39, −0.07]; *p* = 0.005).

The remaining 24 hospitals in South Carolina reported AU to the NHSN for ≥3 years during the 5-year study period. Twenty-two of those hospitals had a publicly reported CDI SIR in both 2017 and 2021. The CDI SIR declined from 0.89 to 0.33 between 2017 and 2021 in those hospitals (mean difference −0.56 [95% CI −0.77, −0.35]; *p* < 0.001).

The decline in CDI SIRs was significantly greater in hospitals reporting AU for ≥3 years than those reporting AU for <3 years (mean difference −0.33 [95% CI −0.57, −0.06]; *p* = 0.016).

## 4. Discussion

From 2017 to 2021, there was an increase in the number of South Carolina hospitals reporting AU to the NHSN, and when AU reporting increased, the overall CDI SIR decreased. The decline in the CDI SIR was significantly greater in hospitals reporting AU for ≥3 years than in hospitals reporting AU for <3 years.

The overall decline in the CDI SIR in South Carolina hospitals is great news for the state. A previous study demonstrated a substantial burden of CDI on the state population and healthcare systems [9]. It is promising that the overall decline in the CDI SIR may reduce the morbidity and cost of hospital-onset CDI in South Carolina. There are multiple possible explanations for the overall decline of CDI SIRs in South Carolina hospitals. First, the CDI SIR is a publicly reported metric that has major financial implications for hospitals, particularly related to reimbursement from the Centers for Medicare and Medicaid Services. There is shared interest between hospital leadership, safety and quality committees, infection prevention, and antimicrobial stewardship programs to improve this metric. It is likely that most hospitals in the state implemented infection prevention, antimicrobial stewardship, and diagnostic stewardship interventions to reduce hospital-onset CDI. It is also possible that ASC-SC’s efforts in education, collaboration, implementation of best practices, and extension of expertise and resources, where needed, facilitated and improved the local interventions.

The association between AU reporting and a steeper decline in the CDI SIR in this study is very intriguing. Hospitals reporting AU for ≥3 years had a 0.56 decline in CDI SIRs between 2017 and 2021 compared to a decline of 0.23 in hospitals reporting AU for <3 years. That constitutes a 0.33 additional decline in CDI SIRs in hospitals consistently reporting AU. These results may encourage more hospitals in South Carolina and nationwide to report AU to the NHSN.

Reporting AU to the NHSN and regularly reviewing institutional SAAR data bring awareness to local antimicrobial stewardship programs and alert hospital leadership to potential areas for improvement. For example, certain hospitals or units within a hospital may have excessive overall or broad-spectrum AU based on high SAAR data. Standardization of AU through the SAAR metric is key to providing an objective comparison of a hospital to their peers with comparable size and patient complexity. Once the reality sinks in that an institution is overutilizing broad-spectrum antimicrobial agents compared to their peer hospitals nationwide, antimicrobial stewardship interventions are developed and implemented to reduce AU. The reduction in AU of broad-spectrum agents likely contributes to the decline in the incidence rate of hospital-onset CDI. This hypothesis is supported by a previous study that demonstrated a reduction in overall and broad-spectrum AU in association with AU reporting and monitoring [10]. In that study, reporting AU to the NHSN and quarterly reviews of SAAR data at a multidisciplinary antimicrobial stewardship subcommittee were beneficial for identifying antimicrobial stewardship opportunities in a multihospital healthcare system, leading to reductions in overall and broad-spectrum AU [10].

There are multiple examples in the literature of a reduction in the CDI SIR through a variety of antimicrobial stewardship interventions. One study demonstrated that requiring prior authorization and a rationale for tier 1 (broad-spectrum) antimicrobial agents, as well as a consultation with infectious disease, was associated with a significant decrease in the CDI SIR [11]. In another study, a reduction in the use of broad-spectrum antipseudomonal agents through syndrome-specific interventions was associated with a significant decline in the incidence rate of hospital-onset CDI in a multihospital healthcare system [12]. In addition, synergistic efforts between infection prevention and antimicrobial stewardship programs have resulted in an even greater decline in the CDI SIR [13]. On the other hand, it also remains possible that hospitals with the resources, time, technical skills, and interest to report AU to the NHSN had more robust antimicrobial stewardship programs overall and were more capable of designing and implementing successful interventions to reduce the CDI SIR. However, this assumption is not supported by the higher CDI SIR at baseline in 2017 in these hospitals compared to others with supposedly less advanced antimicrobial stewardship programs (0.89 vs. 0.69).

To our knowledge, this is the first study to report an association between AU reporting and a reduction in the CDI SIR. The inclusion of a relatively large number of hospitals in South Carolina represents the main strength of the study. This allows for the assessment of statewide collaboration through ASC-SC to achieve a shared goal between hospitals in the state, which is a reduction in the CDI SIR. The inclusion of hospitals from multiple states in future studies may improve the generalizability of the results.

While it is vital to describe the association between AU reporting and CDI SIRs, it is important to acknowledge limitations of this study, as well. Specific hospital characteristics, such as bed size, urban vs. rural, or level of antimicrobial stewardship (in-person, no stewardship, tele-stewardship), were not recorded. Furthermore, details on local antimicrobial stewardship interventions implemented to reduce CDI were not understood at each hospital. Of note, AU in the state of South Carolina during a portion of this study period has previously been published showing a disruption in antimicrobial stewardship during the COVID-19 pandemic [14]. Diagnostic stewardship interventions were also not captured, such as the type of CDI diagnostic test used or institutional limitations to the ordering of CDI laboratory tests, which have been previously demonstrated to reduce hospital-onset CDI rates [15,16]. Lastly, we did not test for correlation in this study, but we hope that this is hypothesis-generating for future investigations.

## 5. Conclusions

Statewide antimicrobial stewardship collaborations are essential to improve AU reporting to the NHSN. The association between consistent AU reporting and the decline in the CDI SIR promotes antimicrobial stewardship efforts and encourages more hospitals to report AU.

## Data Availability

Data will be made available upon request from the corresponding author after signing a data sharing agreement.

## Abbreviations

The following abbreviations are used in this manuscript:

AU	antimicrobial use
NHSN	National Healthcare Safety Network
CDI	*Clostridioides difficile* infection
SIR	standardized infection ratio
SAAR	standardized antimicrobial administration ratio
ASC-SC	Antimicrobial Stewardship Collaborative of South Carolina
